# SKIP Downregulation Increases TGF-*β*1-Induced Matrix Metalloproteinase-9 Production in Transformed Keratinocytes

**DOI:** 10.6064/2012/861647

**Published:** 2012-08-02

**Authors:** Jelena Kocić, Victor Villar, Aleksandra Krstić, Juan F. Santibanez

**Affiliations:** ^1^Laboratory for Experimental Hematology, Institute for Medical Research, University of Belgrade, Dr. Subotića 4, P.O. Box 102, 11129 Belgrade, Serbia; ^2^Department of Biology, University of the Balearic Islands, Ctrretera Valldemossa, Km 7.5, 07122 Palma de Mallorca, Spain; ^3^Laboratorio de Biología Celular, Instituto de Nutrición y Tecnología de los Alimentos, Universidad de Chile, Santiago, Chile

## Abstract

Transforming growth factor-beta (TGF-*β*1) is a potent inductor of matrix metalloproteinase-9 (MMP-9) in transformed cells. Recently, Ski-interacting protein (SKIP) has been described as a regulator of TGF-*β*1 signal transduction, but its role in the induction of cell malignance by TGF-*β*1 has not been fully elucidated so far. In the present study, we analyzed the role of SKIP on TGF-*β*1-induced MMP-9 production. Mouse transformed keratinocytes (PDV) were stably transfected with SKIP antisense construct. We observed that SKIP depletion provoked an enhancement in the expression of MMP-9 in response to TGF-*β*1 treatment. The downregulation of SKIP produced an enhancement in TGF-*β*1-activated ERK1,2 MAP kinase as well as increased transactivation of downstream Elk1 transcription factor. The increased MMP-9 production in response to TGF-*β*1 was dependent of MAPK activation as PD98059, an MEK inhibitor, reduced MMP-9 expression in SKIP antisense transfected cells. Thus, we propose SKIP as a regulatory protein in TGF-*β*1-induced MMP-9 expression acting by controlling ERK1,2 signaling in transformed cells.

## 1. Introduction

The transforming growth factor-beta (TGF-*β*) superfamily of factors is implicated in the regulation of cell proliferation, differentiation, migration, extracellular matrix production, apoptosis, and tumorigenesis [1]. TGF-*β* binds to the functional complex of TGF-*β* family of receptors (T*β*Rs) at the cell surface, which consist of two type II and two type I transmembrane serine/threonine kinase receptors [1, 2]. Receptors, in turn, activate downstream cellular components, including Smads and members of the Ras/MAP kinase pathways [3]. TGF-*β*1 has been postulated to have a dual role in tumour progression, by acting as a tumour suppressor in early stages of carcinogenesis and exerting a prooncogenic role in the last steps of metastatic disease contributing to tumour cell invasion and metastasis [4, 5].

Degradation of the basal membrane and the collagenous extracellular matrix (ECM) presents a critical step in the tumor invasion [6]. Members of the matrix metalloproteinase (MMP) family are involved in the degradation of ECM and implicated in malignancy [7]. Among the human MMPs reported to date, MMP-2 and 9 are the key enzymes involved in degradation of type-I and IV collagen in ECM [8]. Both MMP-2 and 9, which are abundantly expressed in various malignant tumors, contribute to cancer invasion and metastasis [9]. MMP-9 can be stimulated by the inflammatory cytokine tumor necrosis factor (TNF)-∝, by the epidermal growth factor, and by TGF-*β*, through the activation of different intracellular signaling pathways [10]. TGF-*β*1 regulates MMP-9 expression, either by inducing its expression through Ras-ERK MAPK or by the negative regulation of MMP-9 expression related to Smad3 [11, 12]. In fact, it was reported that Smad3 null mice show deregulated expression of MMP-9 [13] while higher expression of this enzyme was detected in serum of osteoarthritis patients with Smad3 mutation [14]. These data suggest a fine regulation of MMP-9 expression by TGF-*β*1 either by Ras-ERKs or Smad3. 

Recently, it has been reported that Ski-interacting protein (SKIP) interacts with Smad2,3 to augment TGF-*β*-dependent transcription [15], suggesting that SKIP may play a role in regulating cell behavior through the TGF-*β* pathway. SKIP was originally isolated as a putative coactivator protein which interacted with VDR and v-Ski protooncogene, using yeast 2-hybrid screening strategies [16, 17]. SKIP is a well-conserved transcriptional adaptor protein that, depending on the cellular context, functions to recruit either activation or repression complexes to mediate multiple signaling pathways involved in the control of cell proliferation and differentiation [18]. However, its precise role in stimulation of tumorigenesis by TGF-*β*1 is poorly understood.

In the present work, we aimed to evaluate the role of SKIP on TGF-*β*1-induced MMP-9 expression and their implications in the regulation of intracellular signal transduction involved. Results obtained revealed that SKIP participates in TGF-*β*1-induced MMP-9 expression in transformed cells. SKIP depletion enhances TGF-*β*1-induced MMP-9 production concomitantly with an increment of TGF-*β*1-activated ERK1,2 signaling, implying the role of SKIP in TGF-*β*1-induced expression of MMP-9 in mouse transformed keratinocytes.

## 2. Material and Methods

### 2.1. Plasmids, Antibodies, and Inhibitors

Mouse pREP4 (antisense mouse SKIP) was kindly provided by Dr. D. Hayward (John Hopkins University School of Medicine, USA). HA-SKIP was kindly provided by Dr. M. Hayman (Stony Brook University, USA). The reporter (SRE)-luc, containing a serum-responsive element, was provided by Dr. A. Corbí (Centro de Investigaciones Biológicas, Madrid, Spain). The reporter system p-FA2-Gal4-Elk1/pFR-luc containing an Ets-like transcription factor Elk1 activation domain that confers MAPK specificity was purchased from Stratagene (La Jolla, CA, USA). Phospho-p44/42 MAPK (Thr202/Tyr204) mouse mAb was from Cell Signalling Technology, Inc. (Danvers, MA, USA); SKIP rabbit antibody (C-15), ERK1,2 rabbit antibody ((C-16): sc-93), and Smad2/3 ((FL-425): sc-8332) rabbit polyclonal antibodies were purchased from Santa Cruz Biotechnology (CA, USA). Anti-HA, anti alpha-tubulin, and secondary antibodies coupled to horseradish peroxidase were purchased from Sigma (Saint Louis, MO, USA). The p-Smad3 rabbit polyclonal antibody and the MEK1,2 inhibitor PD98058 (used at 25 *μ*M) were purchased from Calbiochem, (Darmstadt, Germany). Human recombinant TGF-*β*1 was from R&D Systems (Minneapolis, MN) and used as indicated by manufacturer.

### 2.2. Cell Culture and Transfection Procedures

The transformed mouse keratinocyte cell line (PDV) was kindly provided by Dr. M. Quintanilla (IIB-CSIC, Spain) and cultured as described previously [11]. For stable transfections, PDV cells (~10^6^) seeded in 60 mm plates were transfected with 5 *μ*g of either antisense mouse-SKIP plasmid or empty pCDNA3 vector using Superfect (Qiagen, Hilden, Germany) following the manufacturer's instructions. Transfected cells were selected by growing in medium containing 10% fetal bovine serum and 400 *μ*g/mL of G418 for two weeks. Individual clones were isolated by cloning rings. 

Transient transfections to analyze MMP-9 promoter activity, Gal4-Elk1 and SRE-luc, were performed as previously described [11, 19]. Firefly luciferase activity (Promega, Adison, WI, USA) was standardized for *β*-galactosidase activity (Tropix, Bedford, MA, USA).

### 2.3. Western Blot, Zymography, and RT-PCR Assays

Western blots were performed as described elsewhere [11]. Gelatinase activity was assayed in serum-free medium conditioned for 24 h in cell cultures treated or not with TGF-*β*1. Conditioned media were subjected to SDS-PAGE zymography in gels containing 1 mg/ml gelatine, as reported by Santibáñez et al., 2002 [11].

Total RNA was obtained using Trizol and complementary DNA was generated by the SuperScript First-Strand Synthesis System for RT-PCR (Invitrogen, Carlsbad, CA, USA) using oligo (dT) primer. The following primers were used in this study: *mouse MMP-9*: 5′-ACC-ACC-ACA-ACT-GAA-CCA-CA-3′ forward, 5′-ACC-AAC-CGT-CCT-TGA-AGA-AA-′3 reverse, 304 bp; and *GADPH*: 5′ACC-ACA-GTC-CAT-GCC-ATC-AC 3′ forward, 5′TCC-ACC-ACC-CTG-TTG-CTG-TA 3′ reverse, 452 bp. Products were obtained after 30–35 cycles of amplification and separated by electrophoresis in 1.2% agarose gels.

### 2.4. Statistics

Data are given as means (±SEM) from at least three independent experiments. Asterisks (∗) denote significant differences at a value of *p* < 0.05 for experimental groups being compared with control in the absence of TGF-*β*1, while “&” denotes significant differences at value of *p* < 0.05 for experimental groups being compared with cells in the presence of TGF-*β*1 only, as determined by Student's *t*-test.

## 3. Results

### 3.1. Effect of TGF-*β*1 on MMP-9 Expression and Smad3/ERK1,2 Activation in PDV Cells

**Figure 1:**
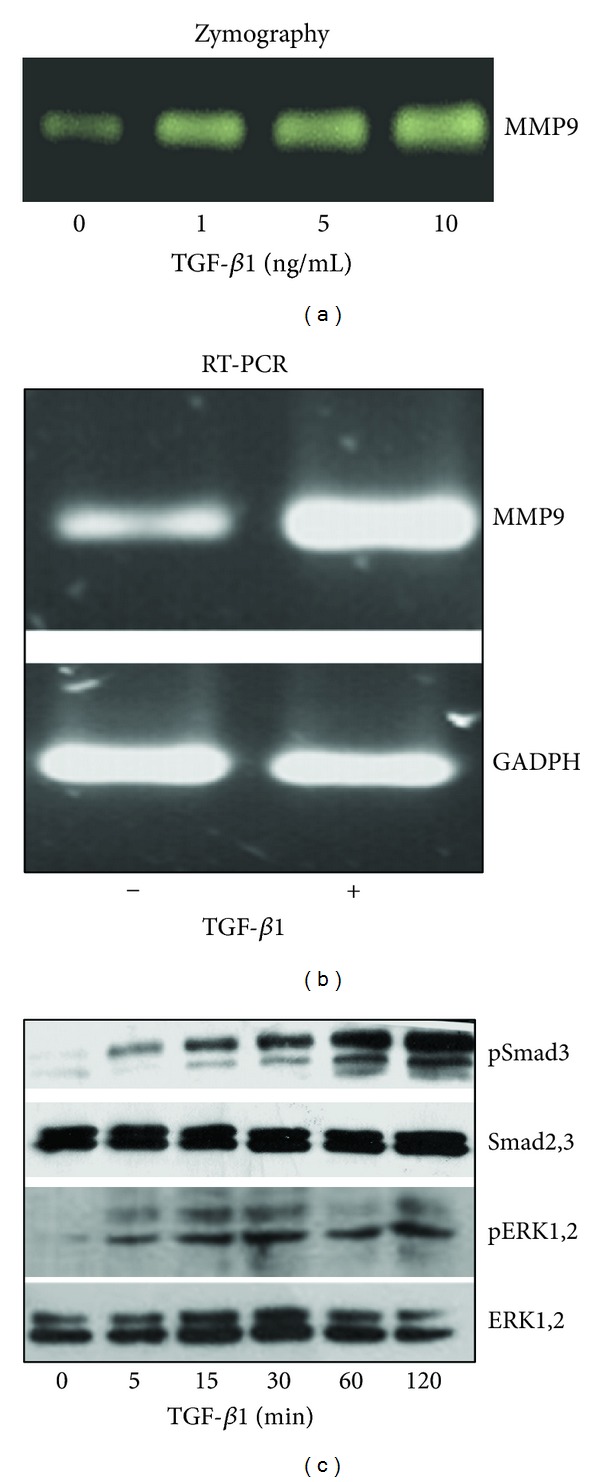
TGF-*β*1 induces MMP-9 expression and activates Smad3 and ERK1,2 signaling in transformed keratinocytes, PDV. (a) Zymography analysis of MMP-9 in serum-free conditioned media. PDV cells were treated for 24 h with different concentrations of TGF-*β*1; the media collected were subjected to electrophoresis on a gelatin-polyacrylamide gel. (b) RT-PCR analysis of MMP-9 expression. PDV cells were treated for 24 h with 5 ng/ml of TGF-*β*1, and mRNA obtained was reverse transcribed to cDNA. PCR products were separated in agarose gels. GADPH was used as housekeeper gene to verify the equal amount of cDNA in samples. (c) Western blot analysis for TGF-*β*1-induced activation of Smad3 and ERK1,2 MAPK. Cells were treated with 5 ng/ml of TGF-*β*1 at times indicated. The samples were assayed for phosphorylated forms of Smad3 and ERK1,2. The detection of total forms of Smad3 and ERK1,2 were used to confirm the amount of each protein loaded.

To determine the MMP-9 activity after TGF-*β*1 treatment, serum-free conditioned media from PDV cells were assayed by gelatin zymography. As shown in Figure 1(a), zymography revealed that TGF-*β*1 stimulates the production of a 92-kDa MMP-9 protein in a dose-dependent manner in PDV cells treated with increasing concentrations of TGF-*β*1 (1, 5, and 10 ng/ml) for 24 hours. Semiquantitative RT-PCR analysis revealed that in PDV cells MMP-9 mRNA was upregulated after 24 hours (Figure 1(b)) of treatment with TGF-*β*1 (5 ng/ml) compared with constantly expressed levels of GAPDH mRNA, which served as an internal control.

Further on, signaling pathways activated by TGF-*β*1 were investigated. Phosphorylated ERK1,2 and Smad3 proteins were detected in PDV cells by western blot using phospho-specific polyclonal antibodies. As shown in Figure 1(c), western blot revealed that 5 ng/ml of TGF-*β*1 activated the phosphorylation of ERK1,2 as early as 5 minutes and peaking at 30 minutes during the time course tested. Also, TGF-*β*1 induced the phosphorylation of Smad3 after 5 minutes with maximum activation at 120 minutes.

### 3.2. SKIP Depletion Enhances TGF-*β*1-Induced MMP-9 Expression/Secretion

**Figure 2:**
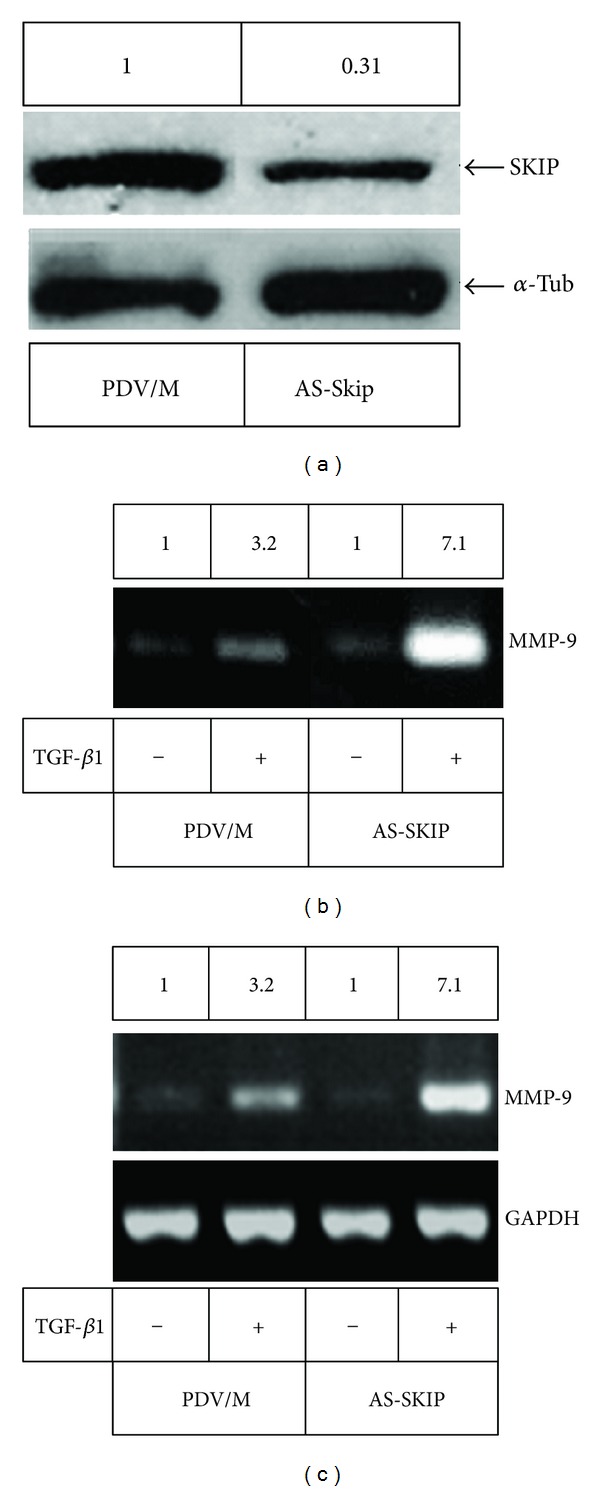
SKIP depletion enhances TGF-*β*1-induced MMP-9 expression/secretion. (a) Antisense depletion of SKIP in PDV cells determined by western blot. PDV/M, PDV parental/mock cells transfected with empty plasmid, AS-S, clones transfected with antisense SKIP plasmid, showing reduced SKIP expression. (b) MMP-9 activity was determined by gelatine zymography in the serum free conditioned media of cell transfectants untreated or treated with TGF-*β*1 for 48 h. The activity of MMP-9 produced a clear band in the gel by gelatine degradation. (c) Expression of MMP-9 mRNA transcripts was determined by RT-PCR in the cell transfectants before and after stimulation (24 h) with TGF-*β*1. GAPDH was amplified as control for the amount of cDNA present in each sample.

**Figure 3:**
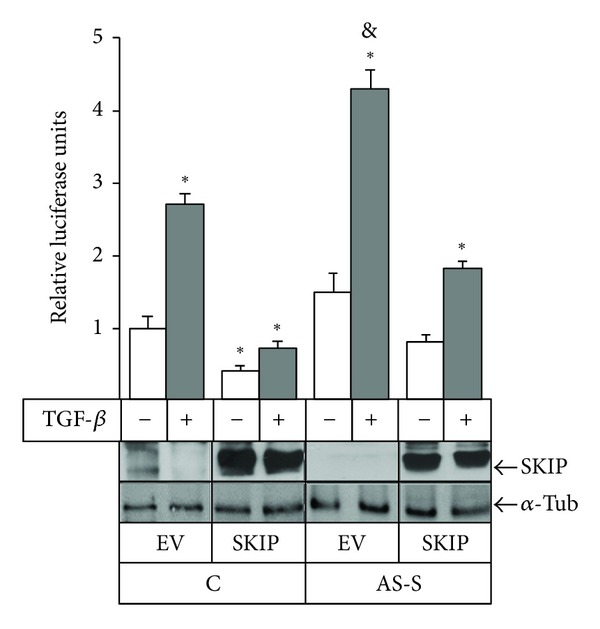
SKIP depletion enhances TGF-*β*1-induced MMP-9 promoter transactivation. Transactivation of MMP-9 promoter in PDV/M and AS-S cells transfected with empty vector (EV) or plasmid containing HA-SKIP. MMP-9 promoter activity was assayed in cells stimulated and not with TGF-*β*1 for 48 h by using luciferase inducible activity. *β*-Gal was used as internal control of transfection. Insert, western blot showing the correct expression of ectopic SKIP. Alpha Tubulin (*α*-Tub) was used a loading control protein.

Given that we have previously shown that TGF-*β*1 promotes expression of MMP-9 in PDV cells [11], our next goal was to analyze whether SKIP modulates TGF-*β*1-induced MMP-9 production. Therefore, PDV cells were stably transfected with a SKIP antisense construction, and the secretion and expression of MMP-9 in these cells were studied.  Figure 2(a) shows a PDV clone with reduced expression of SKIP, designated as AS-S, in comparison with control PDV/Mock (PDV/M) cells, transfected with control vector. As shown in Figure 2(b), and as detected by zymography, stimulation of MMP-9 production by TGF-*β*1 was strongly enhanced in AS-S cells relative to stimulated control cells. This result paralleled with the result obtained by RT-PCR analysis, where the expression of the MMP-9 transcript was enhanced in AS-S cells under TGF-*β*1 treatment compared to PDV/Mock cells (Figure 2(c)). Also, the effect of SKIP depletion on the ability of TGF-*β*1 to transcriptionally stimulate MMP-9 expression was evaluated by expressing a reporter construct containing the luciferase gene under the control of the MMP-9 promoter. It was observed that the lower expression of SKIP in AS-S is correlated with the enhancement of TGF-*β*1-induced transactivation of MMP-9 promoter, whereas the ectopic overexpression of SKIP in control cells inhibited the stimuli of TGF-*β*1 on MMP-9 promoter activity. Moreover, the transfection with SKIP in AS-S also reduced the response of cells to TGF-*β*1 (Figure 3).

### 3.3. Depletion of SKIP by Antisense Strategy Decreases Smad3 and Enhances ERK1,2 Signaling Initiated by TGF-*β*1

**Figure 4:**
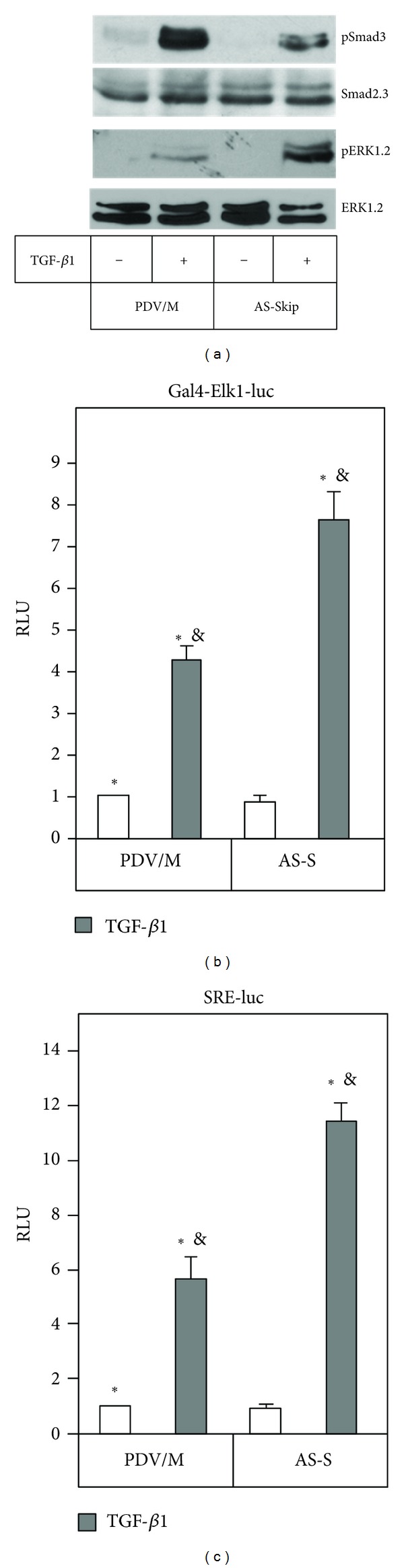
Depletion of SKIP decreases Smad3 and enhances ERK1,2 signaling by TGF-*β*1. (a) Smad3 and ERK1,2 phosphorylation after 60 min of TGF-*β*1 treatment, determined by western blot in PDV/M and AS-S cells. (b) Elk-1-dependent transcription after 24 h of TGF-*β*1 treatment in PDV/M and AS-S cells. (c) Transcriptional activity of ERK1,2 in PDV/M and AS-S cells determined by using SRE-luc reporter. RLU, relative luciferase units.

Considering previous work, which demonstrated that SKIP modulates Smad3 signalling in response to TGF-*β*1 [15], we further analyzed how AS-S cells respond to the activation of signal transduction by TGF-*β*1. Additionally, since TGF-*β*1 induces MMP-9 through ERK1,2 in PDV cells [11], we investigated whether SKIP depletion affects ERK1,2 activation. As expected, the depletion of SKIP in PDV cells (AS-S) decreased TGF-*β*1-mediated induction of Smad3 phosphorylation (Figure 4(a)). Also, significant changes were observed in the TGF-*β*1-induced ERK1,2 phosphorylation levels in AS-S cells in respect to the control. A noteworthy enhancement of downstream ERK1,2 signalling activation by TGF-*β*1 was also noticed, since both transactivation of Elk-1 transcription factor and serum response elements (SRE) was greatly incremented in AS-S cells in response to TGF-*β*1. This result was obtained after cell transfection using the hybrid Elk1-Gal4 and serum response element (SRE-luc) reporter systems (Figures 4(b) and 4(c)).

### 3.4. The TGF-*β*1-Increased MMP-9 Expression in Downregulated SKIP Cells is Mediated by ERK1,2 Signaling

**Figure 5:**
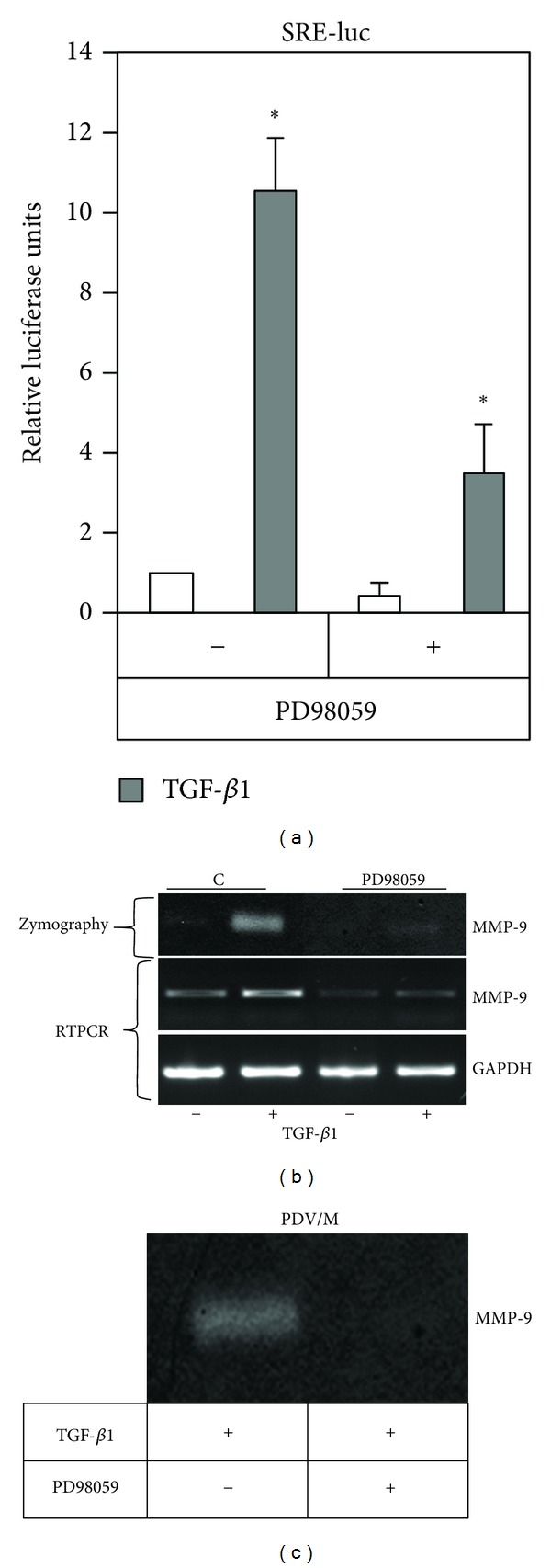
The enhancement of ERK1,2-dependent signaling mediates the TGF-*β*1-increased MMP-9 expression in downregulated SKIP cells. (a) Inhibition of transcriptional activity of ERK1,2-dependent SRE-Luc reporter by PD98059 (25 *μ*M) in AS-S cells. (b) Gelatine-zymography assay and RT-PCR analysis in AS-S cotreated with the MEK1,2 inhibitor PD98059 and/or TGF-*β*1. Cells were treated for 48 h for zymography and 24 h for RT-PCR.* GAPDH* was amplified as RT-PCR control for the amount of cDNA present in each sample. (c) Gelatine-zymography assay of PDV/Mock control cells treated 48 h with TGF-*β*1 in the presence or absence of PD98059.

In order to determine the role of ERK1,2 signalling on the enhancement of TGF-*β*1 induced MMP-9 by SKIP downregulation, experiments using MEK1,2 chemical inhibitor PD98059 were performed. As shown in Figure 5(a), the treatment of AS-S cells with PD98059 drastically inhibited SRE transactivation by TGF-*β*1. The inhibition of ERK1,2 signalling strongly decreased the capacity of TGF-*β*1 to increase MMP-9 production in AS-S determined either by RT-PCR or Zymography analysis (Figure 5(b)). Furthermore, PD98059 inhibited the TGF-*β*1-stimulated MMP-9 in PDV/M control cells (Figure 5(c)). 

## 4. Discussion 

TGF-*β*1 is a potent inductor of MMP-9 production in transformed cells, and although the participation of Smad3 and ERK1,2 in the regulation of TGF-*β*1-induced MMP-9 expression has been reported [11, 12], the modulation mechanisms of this signaling pathway are not well elucidated. In the present work, we analyzed the role of SKIP in the production of MMP-9 induced by TGF-*β*1. We observed that the depletion of SKIP, by antisense strategy, provoked an increment in the production of MMP-9 in response to TGF-*β*1 (Figures 2 and 3). Even though it was reported that SKIP modulates Smad3 in TGF-*β*1 signal transduction [15], there is no data about TGF-*β*1 response genes, such as MMP-9. The results shown here represent new findings, which imply that SKIP is required for the regulation of MMP-9 induction by TGF-*β*1. SKIP is postulated as a negative and/or positive transcriptional regulator and appears to modulate a number of key signaling pathways involved in the control of cell proliferation and differentiation, and as such may play a role in oncogenesis and development. In that way, the regulatory role of SKIP on Smad3 signaling was recently reported [15], and as we also noticed, the down-regulated expression of SKIP provokes low activation of Smad3 after TGF-*β*1 treatment (Figure 4(a)). This is in agreement with this report, even though this study was performed by overexpressing SKIP.

In addition to the known fact that TGF-*β*1 activates a plethoric signal transduction beyond Smads, including ERK1,2 MAPK [3, 20], we previously reported that this growth factor induces the expression of MMP-9 by activating ERK1,2 signaling [11]. The results presented here demonstrate that SKIP reduction produced significant changes in TGF-*β*1-induced ERK1,2 phosphorylation (Figure 4(a)) and also incremented the ERK1,2-dependent activation of downstream signaling initiated by TGF-*β*1. Enhanced activation of the transcription factor Elk-1, also named ETS domain-containing protein Elk-1, as well as enhanced transactivation of the cis-serum response elements (SRE) was produced in AS-S cells in response to TGF-*β*1 (Figures 4(b) and 4(c)). It has been demonstrated that the active phospho-Elk1 binds to the MMP-9 promoter and induces the transcription of MMP-9, as well as that MMP-9 promoter contains SRE Ets box binding sites that are essential for MMP-9 expression [21]. Thus, we may speculate that the depletion of SKIP enhances the capacity of TGF-*β*1-activated ERK1,2 to induce the transactivation of Elk-1-SRE axis, and thereby increase the expression of MMP-9 in cells following TGF-*β*1 treatment. The activation of ERK1,2 is crucial for the MMP-9 production in AS-S cells, as demonstrated by MEK1,2 inhibition with its chemical inhibitor PD98059, which in turn inhibited the transactivation of SRE (Figure 5). Even though SKIP depletion produces a reduction in Smad3 signaling (Figure 4(a)), we did not perform experiments to determine if the solely Smad3 inhibition can mimic SKIP depletion. Interestingly, other authors have reported that both Smad3 and ERK1,2 mediate TGF-*β*1-induced MMP-9 expression [22, 23]. It is possible that the participation of both Smad3 and ERK1,2 in the effect of TGF-*β*1 on MMP-9 expression may be tissue or cell-type specific. In addition, missense mutations of the Smad3 gene and knockout Smad3 mice show an increase in the MMP-9 production [13, 14], implying that in some cases Smad3 may be a negative regulator of MMP-9 expression, although no other signal transduction pathways further than Smad3 were analyzed in these reports. Interestingly, the knockdown of SKIP with iRNA enhances TGF-*β*1-induced MMP-9 in the prostate carcinoma cells PC-3 (Villar et al. unpublished results), suggesting that SKIP is also able to regulate MMP-9 expression in other cancer cells.

Intriguingly, we have reported that SKIP is necessary for the expression of the urokinase type plasminogen activator, uPA, a key proteinase involved in cancer cell invasion and metastasis [24], but conversely to MMP-9, SKIP depletion decreased the induction of uPA by TGF-*β*1, which suggested a differential involvement of SKIP in TGF-*β*-induced cancer proteinases. Additionally, recently we have also reported that Smad3 activity was highly required in the induction of uPA expression by TGF-*β*1 [25], which might explain the divergent role of SKIP on uPA and MMP-9 expression, since in our cell model Smad3 does not play an important role in TGF-*β*1 induced MMP-9 (data not shown). Further studies are necessary to elucidate in deep this differential effect of SKIP. 

Given that SKIP in PDV cells appears to be necessary for the regulation of ERK1,2 signaling, the mechanism underlying the increase of ERK1,2 activation remains to be resolved. Nonetheless, SKIP might participate as a control switch in the fine tuning of signal transduction of TGF-*β*1 in cancer cells, acting as a regulator of Smad3 and/or ERK1,2 signals, by coordinating the magnitude and duration of these signals according to the cellular requirements. In conclusion, our results provide evidence that SKIP mediates TGF-*β*1-induced activation of ERK1,2 downstream signaling, resulting in an enhancement of TGF-*β*1-induced MMP-9 expression in epidermal transformed keratinocytes.

## Abbreviations

TGF-*β*1: transforming growth factor-*β*1
SKIP: Ski-interacting protein
MMP-9: Matrix Metalloproteinase-9
MAPK: Mitogen-activated protein kinases.

## References

[1] Shi Y, Massagué J (2003). Mechanisms of TGF-*β* signaling from cell membrane to the nucleus. *Cell*.

[2] Dennler S, Goumans MJ, Dijke PT (2002). Transforming growth factor *β* signal transduction. *Journal of Leukocyte Biology*.

[3] Derynck R, Zhang YE (2003). Smad-dependent and Smad-independent pathways in TGF-*β* family signalling. *Nature*.

[4] Roberts AB, Wakefield LM (2003). The two faces of transforming growth factor *β* in carcinogenesis. *Proceedings of the National Academy of Sciences of the United States of America*.

[5] Wakefield LM, Roberts AB (2002). TGF-*β* signaling: positive and negative effects on tumorigenesis. *Current Opinion in Genetics and Development*.

[6] Woodhouse EC, Chuaqui RF, Liotta LA (1997). General mechanisms of metastasis. *Cancer*.

[7] Deryugina EI, Quigley JP (2006). Matrix metalloproteinases and tumor metastasis. *Cancer and Metastasis Reviews*.

[8] Westermarck J, Kähäri VM (1999). Regulation of matrix metalloproteinase expression in tumor invasion. *FASEB Journal*.

[9] Liabakk NB, Talbot I, Smith RA, Wilkinson K, Balkwill F (1996). Matrix metalloprotease 2 (MMP-2) and matrix metalloprotease 9 (MMP-9) type IV collagenases in colorectal cancer. *Cancer Research*.

[10] Reuben PM, Cheung HS (2006). Regulation of matrix metalloproteinase (MMP) gene expression by protein kinases. *Frontiers in Bioscience*.

[11] Santibanez JF, Guerrero J, Quintanilla M, Fabra A, Martínez J (2002). Transforming growth factor-*β*1 modulates matrix metalloproteinase-9 production through the Ras/MAPK signaling pathway in transformed keratinocytes. *Biochemical and Biophysical Research Communications*.

[12] Wu YP, Feng Y, Yang X, Huang CF (2002). TGF-*β*1 mediated by SMAD3 inhibits the expression of MMP9 in COS7 cells. *Chinese Journal of Biochemistry and Molecular Biology*.

[13] Bonniaud P, Kolb M, Galt T (2004). Smad3 null mice develop airspace enlargement and are resistant to TGF-*β*-mediated pulmonary fibrosis. *Journal of Immunology*.

[14] Yao JY, Wang Y, An J (2003). Mutation analysis of the Smad3 gene in human osteoarthritis. *European Journal of Human Genetics*.

[15] Leong GM, Subramaniam N, Figueroa J (2001). Ski-interacting protein interacts with Smad proteins to augment transforming growth factor-beta-dependent transcription. *Journal of Biological Chemistry*.

[16] Dahl R, Wani B, Hayman MJ (1998). The Ski oncoprotein interacts with skip, the human homolog of Drosophila Bx42. *Oncogene*.

[17] MacDonald PN, Dowd DR, Zhang C, Gu C (2004). Emerging insights into the coactivator role of NCoA62/SKIP in Vitamin D-mediated transcription. *Journal of Steroid Biochemistry and Molecular Biology*.

[18] Folk P, Půta F, Skružný M (2004). Transcriptional coregulator SNW/SKIP: the concealed tie of dissimilar pathways. *Cellular and Molecular Life Sciences*.

[19] Blanco FJ, Santibanez JF, Guerrero-Esteo M, Langa C, Vary CPH, Bernabeu C (2005). Interaction and functional interplay between endoglin and ALK-1, two components of the endothelial transforming growth factor-*β* receptor complex. *Journal of Cellular Physiology*.

[20] Padua D, Massagué J (2009). Roles of TGF*β* in metastasis. *Cell Research*.

[21] Hsieh HL, Wu CY, Yang CM (2008). Bradykinin induces matrix metalloproteinase-9 expression and cell migration through a PKC-*δ*-dependent ERK/Elk-1 pathway in astrocytes. *GLIA*.

[22] Okamoto T, Takahashi S, Nakamura E, Nagaya K, Hayashi T, Fujieda K (2009). Transforming growth factor-*β*1 induces matrix metalloproteinase-9 expression in human meningeal cells via ERK and Smad pathways. *Biochemical and Biophysical Research Communications*.

[23] Sinpitaksakul SN, Pimkhaokham A, Sanchavanakit N, Pavasant P (2008). TGF-*β*1 induced MMP-9 expression in HNSCC cell lines via Smad/MLCK pathway. *Biochemical and Biophysical Research Communications*.

[24] Villar V, Kocic J, Bugarski D, Jovcic G, Santibanez JF (2010). SKIP is required for TGF-*β*1-induced epithelial mesenchymal transition and migration in transformed keratinocytes. *FEBS Letters*.

[25] Kocic J, Bugarski D, Santibanez JF (2012). SMAD3 is essential for transforming growth factor-*β*1-induced urokinase type plasminogen activator expression and migration in transformed keratinocytes. *European Journal of Cancer*.

